# Immune Response to SARS-CoV-2 Infection in Obesity and T2D: Literature Review

**DOI:** 10.3390/vaccines9020102

**Published:** 2021-01-29

**Authors:** Jorge Pérez-Galarza, César Prócel, Cristina Cañadas, Diana Aguirre, Ronny Pibaque, Ricardo Bedón, Fernando Sempértegui, Hemmo Drexhage, Lucy Baldeón

**Affiliations:** 1 Research Institute of Biomedicine, Central University of Ecuador, Quito 170201, Ecuador; jmperez@uce.edu.ec (J.P.-G.); cmcanadas@uce.edu.ec (C.C.); dfaguirre@uce.edu.ec (D.A.); ronny1995rjps@gmail.com (R.P.); 2 Faculty of Medicine, Central University of Ecuador, Quito 170403, Ecuador; rgbedon@uce.edu.ec (R.B.); fersempert@biociencias-ceb.org (F.S.); 3 Hospital Metropolitano, Quito 170509, Ecuador; procelc@gmail.com; 4 Hospital General Docente de Calderón, Quito 170201, Ecuador; 5 Immunology Department, Erasmus Medical Center, 3015 Rotterdam, The Netherlands; h.drexhage@erasmusmc.nl

**Keywords:** SARS-CoV-2, COVID-19, obesity, T2D, innate, adaptive, immunity

## Abstract

In December 2019, a novel coronavirus known as SARS-CoV-2 was first detected in Wuhan, China, causing outbreaks of the coronavirus disease COVID-19 that has now spread globally. For this reason, The World Health Organization (WHO) declared COVID-19 a public health emergency in March 2020. People living with pre-existing conditions such as obesity, cardiovascular diseases, type 2 diabetes (T2D), and chronic kidney and lung diseases, are prone to develop severe forms of disease with fatal outcomes. Metabolic diseases such as obesity and T2D alter the balance of innate and adaptive responses. Both diseases share common features characterized by augmented adiposity associated with a chronic systemic low-grade inflammation, senescence, immunoglobulin glycation, and abnormalities in the number and function of adaptive immune cells. In obese and T2D patients infected by SARS-CoV-2, where immune cells are already hampered, this response appears to be stronger. In this review, we describe the abnormalities of the immune system, and summarize clinical findings of COVID-19 patients with pre-existing conditions such as obesity and T2D as this group is at greater risk of suffering severe and fatal clinical outcomes.

## 1. Introduction

Outbreak of the Coronavirus disease in 2019 (COVID-19) has spread rapidly across the world evidencing the weakness of the public health system worldwide [1,2]. Free access to scientific information related to COVID-19, facilitates the understanding of epidemiological, clinical, and molecular aspects of the new virus, which can help to contain the disease and to give alternatives for treatment [3]. A beta-coronavirus SARS-CoV-2 was identified as the causative of COVID-19. On 11 March due to high spread, infectious potential, morbidity, and mortality, the World Health Organization (WHO) declared COVID-19 as a global pandemic [4]. As of January 2021, over 85 million cases and nearly 2 million deaths worldwide have been reported [5].

The majority of infected patients experience mild to moderate symptoms such as fever, headache, cough, myalgia, and diarrhea [6,7]. However, people living with pre-existing conditions such as obesity, cardiovascular diseases, type 2 diabetes (T2D), chronic kidney and lung diseases, can develop a severe acute respiratory syndrome (SARS), requiring mechanical ventilation and admission to an intensive care unit (ICU) [8,9]. The high prevalence of obesity and T2D around the world reveals a huge public health demand to combat COVID-19 infection. Currently, obesity affects more than 650 million people and 463 million has T2D worldwide [10,11]. Both diseases share a common feature, augmented adiposity associated with a chronic systemic low-grade inflammation which induce dysregulation of the immune system and increasing susceptibility to develop infections [8,12,13,14,15,16]. The pre-existing chronic inflammation in obese and T2D patients with the augmented inflammatory response against the viral infection seems to increase the susceptibility of these patients for developing severe COVID-19.

In this review, we describe the abnormalities of the immune system, and summarize clinical findings of COVID-19 patients of the pre-existing condition immune system such as obesity and T2D. It focuses on the inflammatory state, lymphocytic profile, and predictors of COVID-19 severity, describing the main differences in terms of immune response to SARS-CoV-2 infection when compared to infected healthy individuals.

## 2. How Immune System Fight against Virus

To know how viruses interact with the host immune system is essential for understanding the virulence, pathogenesis, and disease outcomes.

### 2.1. Role of Macrophages and Dendritic Cells against Virus

The innate immune response is the host’s first line of defense to prevent and eliminate infection of the invading virus. The early natural immune activation also plays an important role in stimulating the adaptive immune response [17]. When the virus enters the host cells, a signaling cascade is initiated which triggers several mechanism of innate defense such us inflammation followed by a repair phase when the antigen is cleared [18]. The capacity of the innate immune cells to sense danger is essential for well-regulated immune responses. Macrophages (MP) and Dendritic cells (DCs) are the first line cells of the immune system. Activation of MP by viral agents induce these cells to response secreting inflammatory cytokines, which activate other immune cells generating a positive feedback loop of inflammation. However, the exaggerated inflammatory response can induce pyroptosis (a type of cell death) which may ultimately lead to virus induced immune pathology [19]. MP also secrete chemokines to recruit other immune cells to the site of infection to eliminate the pathogen. Once the antigen is eliminated, MP secrete molecules to balance the immune response to induce tissue regeneration. DCs play a key role in both innate and adaptive immune responses to viral pathogens [20]. There are two main types of DCs, plasmacytoid (pDC) and myeloid (mDC) cells. pDC produce large amounts of type I IFN, which can induce direct antiviral state [21]. mDCs are specialized antigen-presenting cells (APC) which processes the endogenous and exogenous antigens for presentation by major histocompatibility complex (MHC) molecules to T cells through the T-cell antigen receptors (TCR) [18]. MHC-II molecules are expressed on epithelial cells and immune cells such as B cells, monocytes, macrophages and dendritic cells instead MHC class I molecules are expressed in almost all nucleated cells [22]. MHC-I and MHC-II present protein antigen fragments to CD8+ and CD4+ T cells, respectively. Thus, the pattern expression of MHC molecules directs T cells to interact with exactly the right kind of cells. Once mDCs encounters an antigen, they become activated to produce alarm signals in the form of inflammatory cytokines and costimulatory molecules needed to stimulate host cells [18,23,24]. Another important component of the innate immune system key to fight against virus, are the pattern recognition receptors (PRRs) (e.g., Toll-like, NOD and RIG-I receptors), which are engaged to detect viral RNA or DNA to induce pro-inflammatory cytokines and type I interferons (IFNs) in the infected and other immune cells to eliminate the invading virus [25,26,27]. If the virus cannot be eliminated by natural immune responses, the adaptive immune system uses innate signals to help initiate and enhance its activation [28].

### 2.2. Role of CD8+ T Cells against Virus

To become an effector cell, a naïve CD8+ T cell must receive 3 signals to trigger efficient immune responses [29,30]. Once naïve CD8+ T cells recognize foreign antigens presented to them, through the major histocompatibility complex class I (MHC-I); they initiate adaptive immune responses, specifically against these antigens using a unique antigen-binding site in the CDR3 domain of the TCR receptor [31,32,33]. This antigenic stimulation (signal 1) induces T cell division to give rise to a clone of cells with identical specificities [31,34,35]. The second stimulation (signal 2) is generated through the engagement of costimulatory molecules, namely B7-1 (CD80)/B7-2 (CD86) in APCs and CD28 in T cells [36,37,38]. In case, B7 molecules recognize the cytotoxic T lymphocyte antigen 4 (CTLA-4), instead of CD28, the opposite functional effect occurs, thus inducing inhibition [39,40]. Another costimulatory molecule, crucial for CD8+ CTLs activation and memory formation is CD40 [41,42]. The interaction of CD40-expressing CD8+ T cells and CD40L of CD4+ T helper (Th) cells induce activation of CD8+ T cells directly [26,42,43]. Thus, CD8+ T cell responses to all antigens require CD40 signaling. Third, CD8+ T cells receive specific cytokine signals (signal 3), which further enhance, modify, and skew the responding effect [34,35,38]. The described stimulatory signals take place in the formed immunologic synapse, surrounded also by rings of engaged accessory integrin molecules [44,45]. If one of the signals is not present, tolerance occur instead [46]. Once activated, CD8+ T cells become cytotoxic cells (CTL) which are critical to fight against virus. CTLs exercise their cytotoxicity mediated function through the release of granzymes and perforins, FAS ligand/tumor necrosis factor inducing pathways to kill the infected target cell by apoptosis [18,47,48]. After CTLs perform their “effector” function, the cell population contracts and the remaining cells constitute a long-lived memory CD8+ T-cell pool that protects from secondary infection [49,50,51]. Virus can directly infect DC, which theoretically allow direct presentation of viral antigens to CD8+ T cells, but many viruses target other cells than DC, for example lung epithelial cells and thus the host depends on the cross-presentation of viral antigens by DC to activate virus-specific CD8+ T cells [52,53]. The DCs subsets involved in cross-presentation differ depending on the site of infection and the inflammatory environment [54]. Only when DCs are activated, they can trigger effective CD8+ T-cell responses inducing activation and memory formation [55].

### 2.3. Role of CD4+ T Cells against Virus

CD4+ T cells also have a crucial role in antiviral immune responses. Evidence suggests that in cells where viruses replicate (e.g., lung epithelial cells), a number of viral proteins found in the cytoplasm, can load recycled MHC class II molecules in early endosomes or phagosomes for presentation to naïve CD4+ T cells [56]. However, as defense mechanism, virus can block the induction of class II genes and interfere with the loading of viral peptides [57]. The presentation of viral antigens by APCs (DCs, MP, and B cells) through MHC-II induce activation of naïve CD4+ T cells, which originate T helper (Th) cells with different phenotypes and functions (e.g., Th1, Th2, Th17). The dominant cytokine environment, costimulatory molecules and type of antigen presented, determines the polarized CD4+ T helper formation. Activated CD4+ T cells can help B cells to generate stronger and longer-lived antibody responses, which neutralizing function are key in the defense against virus. CD4+ T cells also can help CD8+ T cell to induce its expansion during a primary immune response facilitating the generation of virus-specific memory CD8+ T cell populations [58]. Additionally, CD4+ Th1-stimulatory cytokines like IL-18 and IL-12 stimulates natural killer cells (NK) to exert their cytotoxicity activity [59,60,61]. Apart from their helper function, evidence from animal models suggests that effector CD4+ T cells can contribute directly to viral clearance secreting cytokines with antiviral activities, and functioning like cytotoxic killing cells [62,63,64]. Finally, it is described that once formed, memory CD4+ T cells have enhanced helper and effector activity that allow them rapidly trigger immune defense mechanisms [56,58].

### 2.4. Role of B Cells against Virus

Conventional B cells are key players in the generation of specific and long-lasting antibodies against virus and require the collaboration of other cells [65]. Naïve B-cell activation occurs after the viral encounter, which can occur directly via antigen recognition through B cell receptor (BCR) or through MHC dendritic cell antigen presentation to B cells in the lymph nodes [66,67]. Thus, MHC class II molecules play an important role in the interaction with B cells to produce antibodies [58,59,68]. Following antigen presentation, B cells activates either extra follicular or germinal centers. Extra follicular B-cell responses will generate short-lived and no memory B cells therefore, much of the early-induced antiviral antibody responses, does not seem to contribute to the long-term response to the virus [65]. On the other hand, germinal center (GC) responses generate high-affinity antibody-secreting plasma cells and long-term memory B cells that ensure sustained immune protection, and rapid recall responses against previously encountered foreign antigens [65]. GC responses are the basis of T cell dependent collaboration, where somatic hypermutation and affinity maturation of B cells occurs. Interestingly, it is described that despite the clearance of viral infection, the germinal centers can persist for many months [69]. The first immunoglobulin produced by plasma cells is the IgM, which has low affinity and a relatively short half-live (around 7 days). IgM secretion peaks appears early and then disappears within a few weeks of infection [69]. If the antigen activated B cells receive collaboration of T helper cells via CD40 molecule, they undergo antibody class switching in the constant region of the immunoglobulin heavy chain to produce antibodies of different isotypes or subtypes (IgG, IgA, or IgE) [70,71]. Those antibodies will conserve the antigenic specificity, but their function will differ depending of its localization. IgG is the principal isotype in the blood and extracellular fluids, whereas IgA is the principal isotype in secretions (intestinal and respiratory tract) [67]. IgG is always monomeric, its secretion peaks around 14 days after infection and decreases over time but do not usually return to baseline levels [65]. Secreted IgG has a central importance to protect against viruses since efficiently neutralize viral antigens [72,73]. In addition, it can opsonize viral antigens for engulfment by phagocytes, participate in antibody-dependent cellular cytotoxicity (ADCC) by NK cells, and activates the complement system to mediated lysis of infected cells. IgA form dimers, is a less potent opsonin, and is a weak activator of complement [74,75]. The affinity of the antigen-binding sites for their antigen is critical for the effectiveness of these antibodies against viral pathogens [65]. Although T and B-lymphocytes function differs from those of innate immunity, the functional pathways largely overlap.

Overall, the innate and adaptive arms of the immune response should be viewed as complementary and cooperating. Its proper function is required for an effective virus clearance and inappropriate activation. In many viral infectious diseases, the principal pathological aspects are not related to the direct action of an aggressor agent, but instead by the “virus’ cytopathic effect” or through “immune cytotoxicity”.

## 3. Immune Alterations in Obesity and T2D

**Figure 1:**
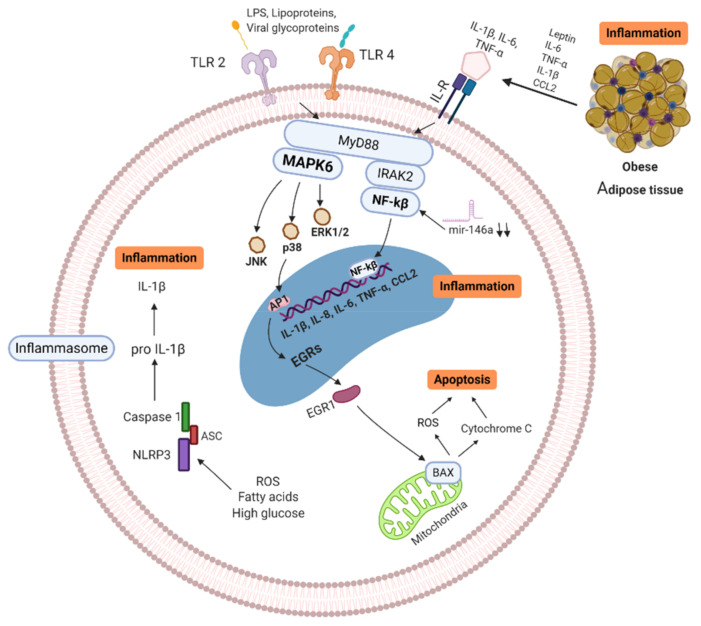
Signaling pathways of obese/T2D immune cell (MNC/MP). Senescence-associated secretory phenotype (SASP) cells secrete high quantity of pro-inflammatory cytokines, which trigger enhanced mitochondrial apoptosis through high mitogen-activated protein kinases (MAPKs). ROS production, free fatty acids and high glucose induce activation of NLRP3 inflammasome, activating IL-1β, leading to further inflammation. In addition, Pattern Recognition Receptors (PRR) and Interleukin Receptors (ILR) signaling through NF-κB and MAPKs pathways, resulting in more inflammatory cytokine production (IL-1β, IL-6, IL-8, IL-18, CCL-2, and TNF-α). Activation of these pathways increases the production of pro-inflammatory cytokines and promotes the infiltration of more pro-inflammatory M1 macrophages in tissues. MAPK: Mitogen-activated protein kinase, IL: Interleukins, ILR: Interleukins receptor, TNF: Tumor necrosis factor, BAX: BCL2-associated X genes, JNK: c-Jun N-terminal kinase, IRAK-2: Interleukin-1 receptor-associated kinase 2, EGRs: Early response genes, NF-κB: nuclear factor kappa B, TLR: Toll-like receptor, CCL2: C-C motif chemokine ligand 2, ROS: Reactive oxygen species, mir146a: microRNA-146a, NLRP3: NLR family pyrin domain containing 3, ASC: Adaptor protein. Created with BioRender.com.

Metabolic diseases such as obesity and T2D share a common feature, augmented adiposity associated with a chronic systemic low-grade inflammation [12,13] which promotes the abnormal production of pro-inflammatory cytokines and the impairment of the immune response and host defense [8]. Adipose tissue is an endocrine organ capable of secreting a diversity of cytokines and adipokines that are involved in the regulation of inflammation and homeostasis [76]. Healthy adipocytes are sensitive to insulin, which is critical for the uptake of glucose and maintenance of blood glucose levels. In obese subjects, adipocytes increase in number and size. This phenomenon is accompanied by an insufficient vascularization of the tissue resulting in hypoxia, triggering apoptosis and/or necrosis as well as elevated secretion of more inflammatory cytokines, adipokines, and chemokines that induce a vast infiltration of immune cells contributing to lipolysis, promoting inflammation and insulin resistance. This metabolic impairment induces the initiation or aggravation of T2D [12]. All these factors generate a low-grade inflammatory microenvironment that incites the recruitment of mast cells, neutrophils, T-cells, B-cells, and the polarization of inflammatory macrophages towards the M1 phenotype is favored in adipose tissue of obese subjects, while maintaining or even reducing the number of regulatory T cells (Treg), T helper cells type 2 (Th2), and M2 macrophages [77]. As a result, the balance shifts from a regulatory anti-inflammatory immune state characterized for the secretion of cytokines IL-4, IL-5, IL-10, IL-13, and IL-33 to an exceedingly inflammatory state secreting multiple proinflammatory cytokines, such as IL-6, IL-8, TNF-α, IL-1β, and chemokine (C-C motif) ligand 2 (CCL2), contributing to maintain a chronic systemic inflammation [6,78]. Additionally, the elevated levels of IL-6 provoke an acute-phase response which increases ferritin, C-Reactive Protein (CRP) and D-dimer [79]. The action of pro-inflammatory cytokines in immune cells can trigger signaling pathways that converge at the NF-κB and MAPK activation, resulting in the generation of intracellular inflammatory cytokine production (IL-1β, IL-6, and TNF-α) and apoptosis induction, respectively. In addition, the increased ROS production induced by excess of fatty acids and high glucose triggers the activation of NLR family pyrin domain containing 3 (NLRP3) inflammasome that, through caspasse-1 activates IL-1β form. Overall, the activation of intracellular pathways accompanied by adipose inflammation increases the production of pro-inflammatory cytokines and promotes the infiltration of more pro-inflammatory M1 macrophages. Additionally, a hallmark in obesity and T2D are the senescent immune cells which exhibit a senescence-associated secretory phenotype (SASP) characterized by secreting a huge quantity of pro-inflammatory cytokines (IL-1β, IL-6, IL-8, IL-18, CCL-2, and TNF-α) [80,81,82]. SASP cells have enhanced mitochondrial apoptosis switching from apoptosis to pyroptosis, through a high mitogen-activated protein kinases (MAPKs) activity which drive IL-1 production (leading to auto-inflammation) and reduced IFN type 1 production, leading to a deficiency to combat viral infections [83] (see Figure 1). The inflammatory cytokines also induce neuro-immune-endocrine interactions affecting the body’s response to stress. Catecholamines secreted by the sympathetic nervous system (SNS) and the adrenal glands are key regulators of metabolism and innate responses which are affected in obesity [84,85]. Animal models of metabolic syndrome have demonstrated the feedback mechanism between inflammatory cytokines and noradrenaline (NA) and the altered phagocytic and microbicidal capacity of macrophages, which caused a greater susceptibility to infections [86,87]. In fact, recent studies have proposed a novel adrenergic regulation of inflammation, via activation of β2-adrenergic receptors, highly expressed in immune cells, to modulate the inflammatory phenotype and activity profile of these cells [85,88].

Besides, important adipokines involved in the inflammatory process are leptin and adiponectin. Leptin is a lipostatic hormone whose main function is to regulate body weight by transmitting signals of satiety to the central nervous system and energy homeostasis [89,90,91]. Evidence shows that leptin is involved in glucose metabolism, innate immune reactions and acute inflammation. The concentration of leptin in blood in obese patients with dyslipidemia is high, causing signaling alteration, promoting the development of insulin resistance and T2D [92,93]. In mice, it has been described an increase in leptin is associated with inflammatory markers in obese individuals [94], and in humans production of cytokines can be induce when leptin is administered exogenously [95]. Adiponectin is a protein whose function is to increase fat oxidation, producing a reduction in the concentration of fatty acids, increasing insulin sensitivity [90,96]. It is described that adiponectin levels decrease in obesity, insulin resistance, T2D and cardiovascular diseases [97]. In addition, alteration in the relationship between leptin and adiponectin ratio has been shown to be associated with insulin resistance [98,99], and in the development of atherosclerosis [100]. Consequently, leptin and adiponectin play an important role in the regulation of cardiovascular and metabolic homeostasis [91,96,101].

MicroRNAs (miRNA) are other important biomarkers that play a relevant role in obesity/T2D [102,103,104]. MiRNAs regulate and modulate the expression of a large number of protein-coding genes [105]. Several studies indicate that miRNA-146a and miRNA-155 are involved in the regulation of inflammatory processes [106,107]. For example, miRNA-155 can promote the activation of the LPS/TNF pathway, thus contributing to the activation of the inflammatory response [108,109]. MiRNA-146a block the nuclear factor kappa B (NF-κB) activation induced by TNF-α and toll-like receptor ligands, therefore its function is crucial in the prevention of excessive immune response [110]. Baldeón L et al. showed that the expression levels of miR-146a in CD14+ in T2D monocytes, was down-regulated when compared to healthy controls. The decreased expression of miR-146a in immune cells, correlates negatively with inflammatory cytokines, thus acting as a negative regulator during the activation of immune responses [111].

Adaptive immune cells (T cells) may play a significant role in the propagation of adipose tissue inflammation in obesity. T cells from obese/T2D patients express lower levels of costimulatory molecules (CD69, CD28, CD40 ligand), and interleukin-12 receptor, as well as, produce lower levels of interferon-γ and granzyme B, compared to healthy individuals [112,113,114]. It was shown in diet-induced obesity mice model that CD8+ T cells infiltrate into fat pads before macrophage infiltration. Additionally, CD8+ T cell depletion with anti CD8+ antibodies, resulted in reduced M1 macrophage infiltrations and decreased inflammatory mediators in adipose tissue, ameliorating insulin resistance and glucose tolerance [115]. Conversely, Winer et al. found that the progression of obesity-associated metabolic abnormalities is under the pathophysiological control of CD4+ T cells. Reconstitution of CD4+ T cells, but not CD8+ T cells, in lymphocyte-free obese Rag1-null mice improved glucose tolerance, enhanced insulin sensitivity, and reduced weight gain [116]. Furthermore, there is evidence that regulatory T cells (Treg) are key regulatory cells in adipose tissue to provide anti-inflammatory signals that block adipose tissue inflammation. Treg cells normally account for 5%–20% of the CD4+ compartment but are thought to be one of the body’s most crucial defenses against inappropriate immune responses [117,118,119]. For example, Feuerer et al. demonstrated that when most of the Treg cells were ablated in fat tissue, proinflammatory transcripts were strongly expressed, suggesting the important anti-inflammatory properties of Treg in metabolic processes [120].

B-lymphocytes have been shown to play a central role in the development of insulin resistance and glucose intolerance by activating CD4+ Th1 and Th17 cells and releasing pathogenic antibodies [121,122]. Epigenetic modifications such as DNA methylation play and important role in the significant increase of B cells proliferation which in turn influence the proliferation of Th17 and the production of proinflammatory cytokines [114,123]. For example, Ip et al. show that in vitroTh17 cells decreased upon depletion of CD19+ cells from PBMCs of T2D patients [124]. In addition, in an animal model it was shown that the lack of mature B cells improved glucose tolerance in HFD mice [114]. Interestingly, posttranscriptional modifications in which various glycosyltransferases are involved are well described to occur in inflammatory disease such as obesity and T2D [125,126]. Wu et al. described that IgG glycosylation profiles were associated with T2D, arguing that glycan score can be used as novel indicator for diabetes and inflammatory status [127].

In conclusion, metabolic disorders such as obesity and T2D, seems to alter the balance of innate and adaptive responses, demonstrated by senescence, exaggerated cytokine inflammation, poor chemotaxis, impaired phagocytosis, glycation of circulating immunoglobulins, and abnormalities in the number and function of CD4+ T and CD8+ T lymphocytes and B lymphocytes.

## 4. Impact of Obesity and T2D in COVID-19 Patients

SARS-CoV-2 entry into the host cells by a spike glycoprotein on the viral envelop via the angiotensin-converting enzyme 2 (ACE2) receptor [128,129]. ACE2, is expressed in a variety of organs and tissues in varying degrees [130]. Studies show that the affinity between the S1 spike receptor-binding domain (RBD) of SARS-CoV-2 and the host ACE2 receptor determines host susceptibility to the virus [131,132,133].

### 4.1. Clinical Characteristics of Obese/T2D Patients with COVID-19

The presence of comorbidities is strongly associated with the development of a severe form of SARS-CoV-2 infection, according to several clinical studies [134,135,136,137]. Once SARS-CoV-2 establishes the disease, it can produce a wide variety of clinical manifestations ranging from asymptomatic to severe symptoms [138]. A retrospective analysis of severe and critical patients reported that 79% had fever, 62% cough, 47% fatigue, 35% dyspnea, 14% joint pain, 12% diarrhea, and 4% headache [139]. Severe disease can occur in healthy individuals of any age but occurs predominantly in elderly adults or those with certain underlying medical comorbidities such as obesity, metabolic syndrome, T2D, or chronic cardiovascular disease [134,135,136,137,140]. In the severe form of the disease, the acute respiratory distress syndrome (ARDS), which is more intense approximately at the second week, there is a need for ventilator support or assisted respiration to overcome the patient’s inability to carry out adequate gas exchange [12,141]. Once severe respiratory failure is established, hemodynamic instability can be triggered (arterial hypotension) and could produce renal failure [142]. Furthermore, vascular alteration of vital implications has been reported, with localized or systemic repercussions, such as a predisposition to develop thrombi and their migration to different organs [143,144]. This further deteriorates the pulmonary circulation of small and large vessels, aggravating respiratory distress due to intrapulmonary thrombosis or thromboembolism, rendering the therapeutic measures established in intensive care units useless where mortality rises up to 40% [145,146,147].

There is a growing body of evidence that hyperglycemia can increase the incidence and severity of viral infections. For example, in a group of morbid obesity patients, approximately 90% had T2D which constitute an independent risk factor for severe influenza virus infection [148]. During severe viral infection, overexpression of adhesion molecules and proinflammatory molecules is thought to impair pulmonary function by allowing the uncontrolled extravasation of leukocytes in the alveolus, which could contributes to the disease pathogenesis observed in COVID-19 [149,150,151,152]. There is evidence that the fluctuations in blood glucose levels known as glycemic variability (GV) has correlation with the endothelial dysfunction T2D patients, characterized by increased expression of adhesion (ICAM-1, VCAM-1) and proinflammatory molecules (IL-8, NF-κB) [149,153,154]. Thus, in severe COVID-19 cases, hyperglycemia on hospital admission could be a determining predictor of mortality at day 28 (HR, 2.09, 95% CI, 1.21–3.64) [155]. A retrospective observational study of 193 patients with severe COVID-19, reported that 25% of which were admitted to the intensive care unit had T2D and required more frequent mechanical ventilation, and their mortality rate was higher (HR, 1.53, 95% CI 1.02–2.30; *p* = 0.041) [9]. Milionis et al., speculates that the increase in mortality rate of diabetic patients with COVID-19, could be related to the sustained activation of chronic inflammation [156]. Patients with COVID-19 and uncontrolled diabetes mellitus, defined by HbA1C levels > 6.5%, presented excessive uncontrolled inflammatory responses and hypercoagulable state, that lead to the development of acute respiratory distress syndrome which was associated with longer hospital stay and a greater probability of death compared to well-controlled diabetic patients (HbA1c < 6.5%) [157,158]. Currently, diabetes management goes beyond glycated hemoglobin (HbA1C) test because it does not take into account fluctuations in blood glucose levels known as glycemic variability (GV) which has been shown to have deleterious effects associated with endothelial dysfunction, characterized by increased expression of adhesion (ICAM-1, VCAM-1) and proinflammatory molecules (IL-8, NF-κB) [153,154,159]. Furthermore, T2D was associated with structural alterations in the lung parenchyma, such as thickening of the capillary alveolus membrane, which increased the ventilatory demands caused by COVID-19 infection [160]. A metanalysis indicates that T2D significantly increases the risk of intensive care unit admission (OR: 2.79) as well as mortality (OR: 3.21) [161]. Besides, the fact that both innate and adaptive immunity are dysfunctional in patients with T2D, it is expected that patients with poor glycemic control, have a higher risk of reinfection by SARS-CoV-2 [162].

Managing people with diabetes and COVID-19 is challenging, evidence shows that a tight glucose control could be very helpful. The usefulness of several biomarkers has been suggested to assess/monitor the severity of the disease in these patients. Interestingly, in patients with hyperglycemia and COVID-19 increased D-dimer levels have been found [163,164]. Quin et al., reported that elevated levels of D-Dimer (OR: 1.095, 95% CI: 1.045–1.148, *p* <0.001) in T2D patients were associated with an increased mortality. In addition, blood hemogram tests showed that in COVID-19 patients, there are an increase neutrophil and a decrease lymphocyte count (OR: 0.180, 95% CI: 0.059–0.550, *p* = 0.003) [139,165]. An elevated neutrophil lymphocyte ratio (NLR) was observed in 80% of severe cases [166]. The variation in the NLR values are significantly correlated with the clinical severity in COVID-19 patients [167,168,169]. In uncontrolled diabetic patients NLR value was increased [158,170]. Thus, NLR has a positive predictive value in T2D patients with COVID-19 (10), which showed a significant increase in neutrophil levels (>6.3 × 109 cells/L) and a decrease in lymphocyte levels (<0.8 × 109 cells/L) [155]. Altered platelet-to-lymphocyte ratio (PLR) is also reported as an important predictive value marker, which positively correlate with the susceptibility to develop a critical case in patients with pre-existing conditions [135,171].

### 4.2. Immune Innate Responses of Obese/T2D Patients with COVID-19

**Figure 2:**
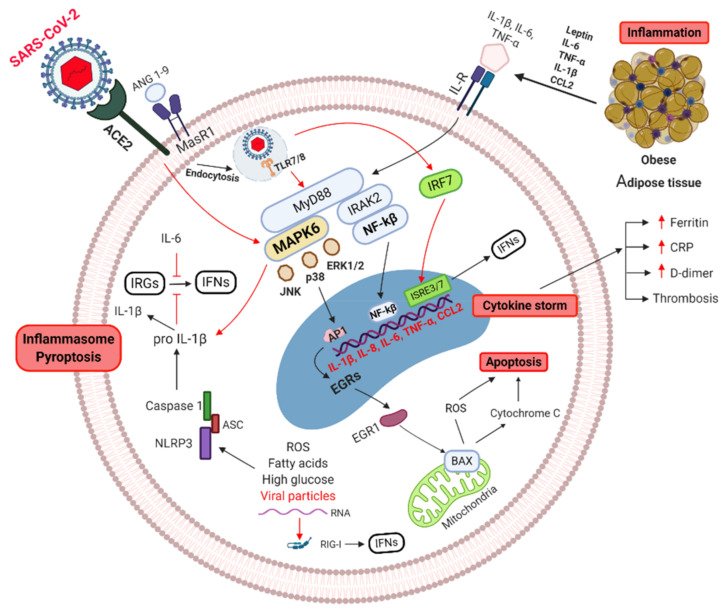
Signaling pathways of obese/T2D cell, of critical COVID-19 cases. Obese and T2D patients have already activated inflammatory pathways. SARS-CoV-2 entry though ACE2 receptor, can alters ACE2/MasR1 axis inducing MAPK6 activation, triggering pyroptosis with high IL-1 production (cytokine storm). Uptake of viral antigens by intracellular Toll Like Receptors (TLR 7/8) can signal through NF-κB pathway, resulting in more inflammatory cytokine production (IL-1β, IL-6, IL-8, IL-18, CCL-2, and TNF-α) (cytokine storm). In addition, viral RNA can induce NLRP3 activation resulting in secretion of IL-1β (inflammasome), leading to further activated inflammation. Excessive inflammatory cytokines (IL-1/IL-6) can block IFN type 1 production. MAPK: mitogen-activated protein kinase, IL: Interleukins, ILR: Interleukins receptor, TNF: Tumor necrosis factor, BAX: BCL2-associated X genes, JNK: c-Jun N-terminal kinase, IRAK-2: Interleukin-1 receptor-associated kinase 2, EGRs: Early response genes, NF-κB: nuclear factor kappa B, TLR: toll-like receptor, CCL2: C-C motif chemokine ligand 2, ROS: Reactive oxygen species, mir146a: microRNA-146a, NLRP3: NLR family pyrin domain containing 3, ASC: Adaptor protein. Created with BioRender.com.

The antiviral innate immune response is the key to contain and eliminate the invading virus. There is evidence that the innate immune response is altered in diseases such as obesity and T2D making people more susceptible to infection [86,88]. Alveolar epithelium cells and MPs are the primary targets of SARS-CoV-2 in the lung [19]. In critical cases of COVID-19, innate cells fail to originate a robust Interferon I and III antiviral response, accompanied by high secretion of chemokines such as CCL2 and IFN-γ-induced protein 10 (IP-10). These molecules are capable to recruit more effector inflammatory cells to the lungs, which secrete abundant proinflammatory cytokines such as IL-6, IL-8, TNF-α, and IL-1β initiating a “cytokine storm”. Several studies documented the interactions between SARS-CoV-2 virus and the hyperactivation of immune cells within the lung, which in turn can trigger a systemic hyperinflammation condition [172,173,174,175,176,177]. The excessively inflammatory activated MP can cause macrophage activation syndrome (MAS), which results in ARDS [178,179]. MAS is directly triggered by cytokines such as IL­6 and TNF-α, and indirectly by IL-1 [172]. The characteristic feature of MAS is a macrophage exhibiting hemophagocytic activity that infiltrate almost any organ causing cytopenia and thrombosis [172,179]. It is speculated that during SARS-CoV-2 infection, the activation of the angiotensin-converting enzyme 2/mitochondrial assembly receptor 1 (ACE2/MasR1) axis in immune cells is down-regulated/dysfunctional, which alters it’s downstream signaling pathway [130]. This can induce activation of MAPK6 pathway triggering the inflammatory cascade leading to a molecular collection of over-regulated pyroptosis with high IL-1 production (cytokine storm) and a reduced production of IFN type 1. In addition, the interferon responsive genes (IRGs) could be down regulated in an IL-1 and IL-6 high environment which predispose to high vulnerability and viral spread [180,181,182,183,184]. In obese/T2D patients infected by SARS-CoV-2, where several inflammatory pathways are already hampered this response appears to be stronger (see Figure 2).

Multiple studies present evidence that correlates the rising of inflammatory cytokines and biomarkers with disease severity. For instance, a retrospective study in Wuhan, China by Wang, W. et al. evaluated 123 COVID-19 patients who were divided into two groups, critical and non-critical. Sixty-five percent of patients had comorbidities including hypertension, diabetes, heart, liver, and respiratory diseases and most of these patients were part of the critical group. Results indicated that C—reactive protein (CRP) and IL-6 positively correlated with the susceptibility to develop a critical case [135]. A similar retrospective study in Japan by Hirashima, T., et al. reported a significant correlation between hypertension, older age, diabetes, and disease severity. The critical group exhibited higher levels of CRP than the moderate group [185]. Consistently, a Spanish retrospective study, showed that IL-6, ferritin, D-dimer, and CRP levels were higher as disease severity increased [186]. Likewise, the authors from another retrospective study found an increase in pro-inflammatory cytokines like IL-6 which showed an inverse correlation to T-lymphocyte count, and these characteristics were also associated with disease outcome and severity [187]. In addition, a retrospective study describes the clinical manifestations and outcomes of 121 cases of COVID-19; 70% of all the cases presented comorbidities, particularly hypertension (40.5%), diabetes (20.7%), and cardiovascular and cerebrovascular diseases (22.3%). The Cox proportional-hazard model indicated that comorbidities are associated with elevated levels of IFN-γ, IL-6, IL10, TNF-α, CRP, and D-dimer [188].

All in all, several reports follow a trend indicating that patients with at least one pre-existing comorbidity (e.g., Obesity and T2D) are more prone to severe form of SARS-CoV-2 infection, and exhibiting elevated levels of inflammatory markers. Most authors suggest the need of special and prompt management with anti-inflammatory therapy to avoid fatal consequences.

An effective innate response and a good regulation of the inflammatory response could prevent the infection from developing completely and, therefore, the low incidence of infection in some individuals and even asymptomatic patients.

### 4.3. Immune Adaptive Responses of Obese/T2D Patients with COVID-19

Adaptive immune cells are responsible for the clearing of viral infections such as the one caused by the SARS-CoV-2. It is expected that pre-existing alterations in adaptive cells (T and B cells) in obese/DM2 patients might lead to a greater risk of having worse clinical outcomes if the SARS-CoV-2 virus infects them. An understanding of the relationship between activation/deactivation of adaptive cells in host response to SARS-CoV-2 is still emerging. Characteristics of T-cell populations during the COVID-19 clinical course seem to follow a clear trend. Most of the clinical studies found in this review compared patients with severe and non-severe symptoms, and survivors vs. non-survivors. By extracting clinical summarized data, various studies showed significant evidence of a systemic reduction in T-cell populations (lymphocytopenia) in obese/T2D patients as COVID-19 progressed [134,155,156,158,162,163,164,170]. Importantly, patients hospitalized with underlying conditions presented worse lymphocytopenia than those who did not report comorbidities [178,185,186,187,189]. Based on this information, the majority of the researchers have proposed that lymphocytopenia could potentially be used as an indicator of COVID-19 severity [190,191,192]. Conversely, to what is reported about the quantitative aspects of T-cell populations during the COVID-19 course, an aspect in which there is a pronounced lack of understanding is whether T-cell response might positively or negatively contribute towards the severity of this disease [193]. In this regard, few studies were found which report that SARS-CoV-2 specific CD4+ T cells were higher in proportion to CD8+ T cells and presented phenotypical and functional alterations in severely affected patients [194,195]. Functionality of SARS-CoV-2–specific CD4^+^ T cells using flow cytometry show that the percentage of cells with the ability to produce IFN-γ, IL-2, and TNF-α was significantly lower in patients with severe courses as compared with convalescent individuals. Besides, it was showed that SARS-CoV-2–specific CD4^+^ T cells from intensive care units patients had significantly higher expression levels of CTLA-4, compared with convalescent patients (*p* = 0.035). In addition, SARS-CoV-2–specific CD8^+^ T cells from UCI patients expressed lower amounts of granzyme B [194].

**Figure 3:**
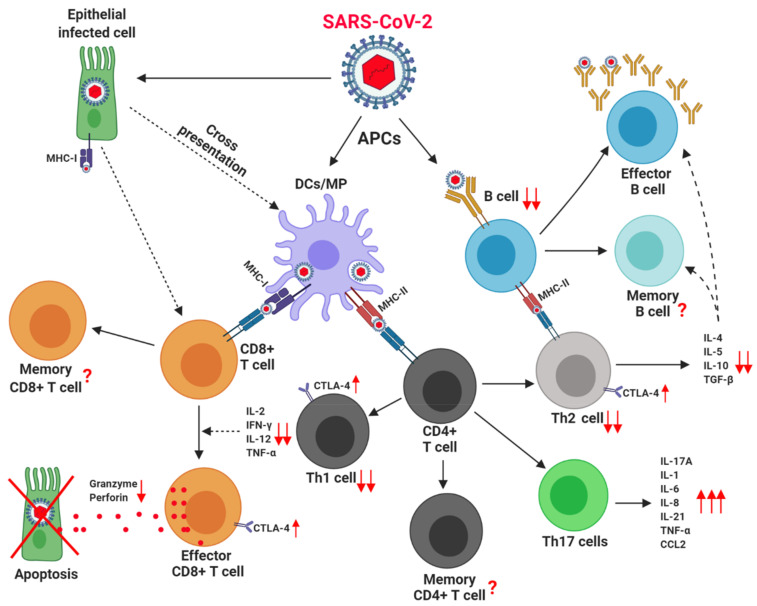
Adaptive Immune cells in obese/T2D patients, with critical COVID-19. Obese and T2D patients have pre-existing adaptive immune cell alterations. SARS-CoV-2 can infect antigen-presenting cells (APC) such as DCs, MP, and B cells, which present viral antigens to T cells via MHC I and MHC-II molecules. MHC-I molecules present cytoplasmic viral protein to CD8+ T cells; while MHC-II molecules present endoplasmic viral proteins to CD4+ T cells. Additionally, SARS-CoV-2 can directly infect other cells (e.g., epithelial cells), which could depend on cross-presentation of viral antigens by DC to activate virus-specific CD8+ T cells. Lymphocytopenia is a hallmark feature of severe COVID-19. The release of cytokines by CD4+ Th1 and Th2 cells appears to be decreased. In contrast, the expression of inhibitory molecule CTLA-4 appears to increase. The frequency and function of Th17 cells appears to predispose the development of ARDS. Despite the low number of B-lymphocytes IgG and IgA specific antibodies seems to be high. APC: Antigen presenting cells, IL: interleukins, TNF: Tumor necrosis factor, IFN: Interferon, DC: Dendritic cells, MP: Macrophages, MHC: Major histocompatibility complex, CTLA-4: Cytotoxic T-Lymphocyte Antigen 4, Th: T helper cells, CCL2: Chemokine (C-C motif) ligand 2. Created with BioRender.com.

Despite the low number of B lymphocytes reported in severe patients, it is described that levels of SARS-CoV-2–specific IgG and IgA antibodies are higher than in convalescent patients [196]. Interestingly, it is not possible to detect specific SARS-CoV-2 antibodies in all infected individuals. Schub et al. reported SARS-CoV-2 IgG specific antibodies in 83% of patients and IgA specific antibodies only in 69% of patients. It is speculated that in severe patients the role of CD4^+^ T cells in providing help for induction of humoral immunity is altered. The percentage of SARS-CoV-2–specific CD4^+^ T cells showed a significant correlation with both specific IgG (*r* = 0.77, *p* < 0.0001) and IgA antibodies (*r* = 0.67, *p* < 0.0001) [194]. Besides, evidence show that critically ill COVID-19 patients have high number and activation of Th17 cells [197,198]. This observation is of particular importance since there is a relationship between IL-17A production and development of ARDS [199,200]. It is hypothesize that increased activity of Th17 cells early in the course of infection with SARS-CoV-2 and subsequent signaling through IL-17A leads to worse clinical outcomes [201] (see Figure 3).

Regarding the loss of specific SARS-CoV-2 antibodies in mild COVID-19, conflicting evidence has appeared. Ibarrondo et al., reported a rapid decay of SARS-CoV-2 antibodies in patients who had recovered from COVID-19 [202]. However, another group report that anti SARS-CoV-2 antibodies remained high until 35 to 40 days after of onset symptoms [203]. In accordance, Bölke et al. reported that IgA levels of antibodies against SARS-CoV-2 remained high until 50 to 60 days after the onset of symptoms and IgG antibodies levels remained elevated, with only a slight decrease, at 120 days after the onset of symptoms [204]. Great concern has been shown as a result of these observations, being necessary to carry out more studies to know if this phenomenon reflects an altered immunomodulation in patients with COVID-19, mainly in those groups with a higher risk of fatal outcome.

## 5. Future Perspectives

It is evident that, obesity and T2D are complex clinical conditions that impairs various key immunological and biological functions, which could prevent vaccine from getting protection in these subjects [205,206,207]. This phenomenon was highlighted since the 2009 pandemic of H1N1 influenza [148,205,208,209]. Reports show that obese people loose specific antibodies steeper than healthy weight people [210,211]. This reduced antibody response is associated with altered activation and function of specific CD4+, CD8+ T lymphocytes and higher levels of intracellular TNF-alpha [206,212]. Studies in animal models also confirm the observation of altered responsiveness of influenza vaccine. Diet-induced obesity mice, showed decreased antibody neutralization capacity [213,214] and obesity induced, failure to maintain influenza-specific CD8^+^ memory T cells, therefore a reduced protective efficacy against H1N1 vaccine [215].

The evidence of diminish virus vaccine effectiveness in obese/T2D patients raise concern about COVID-19 vaccine responsiveness in these population. However, since testing of vaccines has included subjects of these populations it is expected to have them protected.

## 6. Conclusions

There has been great interest in the innate and adaptive immune response of patients with already weakened or unbalanced immune systems as those found in persons suffering from comorbidities related to metabolic syndrome (overweight, obesity, hypertension, T2D). A common feature in these patients is a chronic and low-grade inflammatory state and altered immune function, which appears to increase the risk of having a poorer clinical outcome compared to healthy individuals during the current COVID-19 infection. In this review, we present evidence generated by several research groups that have tried to describe the immune pathological mechanisms that may be responsible for immune dysfunction in obese and T2D patients.

## Data Availability

Not applicable.
